# Perception and attitudes towards preventives of malaria infection during pregnancy in Enugu State, Nigeria

**DOI:** 10.1186/s41043-015-0033-x

**Published:** 2015-11-18

**Authors:** Nkechi G. Onyeneho, Ngozi Idemili-Aronu, Ijeoma Igwe, Felicia U. Iremeka

**Affiliations:** 1 Department of Sociology/Anthropology, University of Nigeria Nsukka, Nsukka, 410001 Nigeria; 2 Humanities Unit, School of General Studies, University of Nigeria, Enugu Campus, Enugu, Nigeria

**Keywords:** Perception, Attitude, Malaria-in-pregnancy, Insecticide treated bednets, Intermittent presumptive treatment, Nigeria

## Abstract

**Background:**

The objective of this study is to explore and document perceptions and attitude associated with uptake of interventions to prevent malaria in pregnancy infection during pregnancy in Enugu State, Nigeria.

**Methods:**

This is a cross-sectional study in three local government areas in Enugu State to identify the people’s perceptions and attitudes towards sleeping under insecticide-treated bednets and uptake of recommended doses of intermittent presumptive treatment during pregnancy. In-depth interview guides were employed to collect data from health workers and mothers who delivered within 6 months preceding the study, while focus group discussion guides were employed in collecting data from grandmothers and fathers of children born within 6 months preceding the study.

**Results:**

The people expressed fairly good knowledge of malaria, having lived in the malaria-endemic communities. However, some were ignorant on what should be done to prevent malaria in pregnancy. Those who were aware of the use of insecticide-treated bednets and intermittent presumptive treatment during pregnancy however lamented the attitude of the health workers, who make access to these interventions difficult.

**Conclusions:**

Efforts to prevent malaria in pregnancy should focus on providing health education to pregnant women and their partners, who reinforce what the women are told during antenatal care. The attitude of health workers towards patients, who need these interventions, should be targeted for change.

## Background

Malaria remains a major challenge to attaining the health-related Millennium Development Goals and of public health in Nigeria, where half of the population suffers at least one episode of malaria in a year. Nigeria, where the disease accounts for 11 % of maternal mortality and 12–30 % of mortality in children under 5 years of age [1], is one of the hardest hit countries in Africa south of the Sahara. Malaria is endemic throughout Nigeria. Fifty percent of the adult population experience at least one malaria episode annually. Prevalence rate of malaria among children under 5 years of age in Nigeria was put at 51.5 % by rapid diagnostic test positive results [2]. Children under 5 years of age may suffer malaria attacks two to four times each year [3]. A study in Abeokuta, in the South-west of Nigeria, found a very high malaria prevalence rate of 62.4 % among women attending traditional birth homes [4]. Health facility prevalence of malaria in pregnancy in Nigeria is estimated at 48 % [5, 6]. The disease currently accounts for nearly 110 million clinically diagnosed cases per year, 60 % of outpatient visits, and 30 % hospitalizations [7]. Mortality range associated with malaria in pregnancy can vary 100-fold across the spectrum of birth weight and rises continuously with decreasing weight [8].

Malaria in pregnancy is also reported to have links with inter-uterine growth retardation [9]. It is estimated to cause up to 15 % of maternal anemia and about 35 % of preventable low birth weight as well as neonatal mortality [10]. Malaria-induced low birth weight is responsible for between 62,000 and 363,000 infant deaths every year in Africa, which translates to 3–17 deaths per 1000 live births [11]. Guyatt and Snow (2004) estimated that malaria in pregnancy caused 11.4 % of neonatal deaths and 5.7 % of infant deaths in malaria-endemic areas of Africa, resulting in approximately 100,000 infant deaths per year [12]. In a study of effects of the use of insecticide-treated bednets on birth outcomes in Southeast Nigeria, Igwe, Ebuchi, Imem, and Afolabi (2007) noted that malaria during pregnancy is also a significant drain on its economy and a major financial burden to the poor [13]. With these indices, achieving the Millennium Development Goals of reducing child and maternal deaths by two thirds and three quarters, respectively, by 2015 in Nigeria will require a more pragmatic approach to address malaria-related morbidity and mortality in Nigeria.

Current efforts to address malaria illnesses and deaths in Nigeria include the adoption of artemisinin-based combination therapy (artemether-lumefantrine and artesunate-amodiaquine) for the treatment of uncomplicated malaria. Furthermore, Nigeria is currently promoting home management of malaria using artemisinin combination therapy in line with the World Health Organization’s recommendation in order to ensure prompt and appropriate access to management of malaria [14]. Sulphadoxine-pyrimethamine is exclusively reserved for preventing malaria during pregnancy. The use of insecticide treated bednets is also highly recommended for protection against contact with *Plasmodium falciparium* parasite. These are contained in the national antimalarial drugs updated 2012 [15].

The use of insecticide-treated bednets has not only remained one of the most important of all measures of protection against malaria; it has become the single most dependable intervention when used properly and efficacious in reducing maternal anemia, placental infection, and low birth weight [16]. Fegan, et al. also reported 44 % reduction in mortality due to insecticide-treated bednet use [17]. In realization of the effectiveness of insecticide treated bednets against malaria, improvements were made in the production of insecticide treated bednets. Between 2008 and 2010, a cumulative total of 289 million insecticide-treated bednets were delivered to sub-Saharan Africa, enough to cover 76 % of the 765 million persons at risk [18].

Despite the tragedy and economic loss caused by malaria, the majority of pregnant women in Nigeria do not have access to these insecticide-treated bednets. In Enugu State, for instance, the NPC and ICF Macro (2009) reports that only 9.7 % of the households surveyed had at least one net while only 3.9 % of the pregnant women slept under a treated net [7]. Reasons for the low utilization may be attributable to a number of socio-cultural factors, including ignorance, poverty, beliefs, and gender issues as well as low utilization of antenatal clinic services among Nigeria women compared to women in other African countries and also the lack of malaria in pregnancy services existing in antenatal clinic’s program [19].

The most promising preventive approach to using antimalarial drugs for pregnant women is intermittent presumptive treatment. Garner et al. estimated that effective prevention of malaria with chloroquine prophylaxis or intermittent presumptive treatment reduces the risk of low birth weight by as much as 43 % [20]. Intermittent presumptive treatment is based on the use of antimalarial drugs given in treatment doses at predefined intervals after quickening [21]. For many years, WHO recommended that pregnant women in malaria-endemic areas should receive an initial antimalarial treatment dose on their first contact with antenatal services, followed by weekly chemoprophylaxis with an effective and safe antimalarial drug [22].

The critical factors responsible for the current levels of utilization of intermittent presumptive treatment and insecticide-treated bednets in preventing malaria in pregnancy in Enugu remain unclear. This paper explored and documented the perceptions and attitude associated with uptake of interventions to prevent malaria in pregnancy infection during pregnancy in Enugu State, Nigeria.

## Methods

### Ethical statement

Ethical approval was received through the health research ethics committee of the University of Nigeria Teaching Hospital. The community leaders and members of the community involved in the study gave their verbal approval for the study. The verbal approval was considered sufficient to avoid a situation where the people will be uncooperative and less outspoken for fear of repercussions.

### Study design

The study was exploratory and adopted a cross-sectional approach using qualitative methods of inquiry, based on in-depth interviews and focus group discussion designs. The study was designed to allow a description and analysis of community perceptions/attitudes towards insecticide-treated bednets and intermittent presumptive treatment in pregnancy sulphadoxine-pyrimethamine for preventing malaria in pregnancy employing qualitative inquiry in Enugu state, Nigeria. Community perception of the interventions is viewed from a perspective that recognizes an interaction among a number of variables, including the socio-demographic realities of the people as well as their past experiences with the health system, health problems, and relevant interventions.

### Study area

The locale for this study was the capital of Enugu State, Enugu Urban. The people of Enugu Urban belong largely to the Igbo ethnic group, which is one of the three largest ethnic groups in Nigeria. The name “Enugu” comes from two Igbo words “enu ugwu” or “top of the hill.” The city’s slogan is “perpetual apex pride.” Nicknamed the “coal city” in the early 1900s, Enugu was a major center for the mining of coal but since the Biafra war, coal production has declined almost to a halt. Thus in recent years, the city’s economy has diversified and is largely dominated by trading, commerce, and small-scale industry as well. A significant proportion of the population is also engaged in the civil service sector.

The major health care facility in Enugu urban is the Enugu State University Teaching Hospital and College of Medicine. There are also numerous private, missionary, and government-owned hospitals and clinics. The people of Enugu also have access to the University of Nigeria Teaching Hospital, located in Ituku Ozalla community of Nkanu West, a neighboring local government area (LGA) to Enugu South LGA.

Enugu urban has three LGA, namely Enugu South, Enugu East, and Enugu North (which is the major business district). Over the years, Enugu urban has grown enormously especially in areas of commerce, estate, and health facilities.

### Study population and sampling

The target population for this study was women within the child bearing age of 15–49 years who gave birth within 6 months before the survey. Their mothers and husbands were also covered in the study. Health care providers were also interviewed. The distribution of data collected in each local government area is contained in Table 1 below.

**Table 1:** Distribution of Sample by LGA and Groups of Study Participants

Groups	Local government area						Total	
	Enugu/E peri urban		Enugu/N urban		Enugu/S rural			
	FGD	IDI	FGD	IDI	FGD	IDI	FGD	IDI
Mothers of women who delivered within 6 months before the survey	4	–	4	–	4	–	12	–
Husbands of women who delivered within 6 months before the survey	4	–	4	–	4	–	12	–
Health workers	–	4		4	–	4		12
Women who delivered within 6 months before the survey	–	6		6		6		18
Total	8	10	8	10	8	10	24	30

A total of nine communities were randomly selected from each LGA. The focus group discussions were equally distributed across local government area. This gave a total of 24 focus group discussions (FGDs) with husbands and mothers of women (15–49 years), who delivered within 6 months before the survey and 30 in-depth interviews (IDI) with health workers and women (15–49 years), who delivered within 6 months before the survey, in the entire study (Table 1). Each focus group discussion session consisted of 8 to 10 participants, selected with the convenience sampling technique, after receiving community consent following social mobilization.

Groups were selected according to participants’ availability. Women who delivered in less than 6 months to the study were equally included in the study based on their availability and willingness to participate in the study. The health workers on the other hand were selected through a two stage sampling. First, 12 health facilities were randomly selected from the list of health facilities in the study LGAs. The officer in charge of the selected health facility was purposively selected for the study.

Before the commencement of the study, the state malaria control officer took the researcher to the community leaders and introduced the mission. The community leaders in turn mobilized the community members and informed them that the officials from the state capital have come to discuss issues relating to their health in the community. They were also informed that some of them will be needed for focus group discussions and another special group for in-depth interviews with the researcher.

### Instrument and method of data collection

The instruments for data collection were focus group discussion and in-depth interview guides, which explored such themes as the common health problems in the communities, knowledge of malaria, practices in preventing malaria in pregnancy, perception of insecticide treated bednets and intermittent presumptive treatment in pregnancy, as well as antenatal care practices for protection against malaria infection during pregnancy. The discussions were tape recorded, where permission was granted. The discussion sessions were made informal with light refreshment served to the participants during discussion, in the case of the focus group discussions. The instruments were pre-tested in one urban and two rural communities in Nsukka local government area, for the sensitivity of the instrument. The pre-testing also provided opportunity for giving targeted orientation on the methods and objectives of the study to the data collectors. FGD and IDI sessions took an average of 45 min and 30 min, respectively.

The team spent approximately 1 week in each local government area for activities which included mobilization of the groups, actual discussion, interviews, and data cleaning. The focus group discussion and interviews were facilitated by social scientists from the Faculty of Social Sciences in the University of Nigeria, which is located within Enugu State. However, the facilitators were also from the same cultural background with the participants in the study.

### Data analysis

Analysis of the data placed emphasis on the interpretation, description, and recording/writing of what was actually said. In going through the transcriptions, phrases with contextual or special connotations were noted and pulled out as illustrative quotes in developing the ethnographic summaries. To do this, relevant themes were developed for the coding and sorting of the qualitative data and Atlas.ti version 5.0 software was used in managing the qualitative data. The transcripts were first done in the local language and translated into English. In going through the transcriptions, phrases with contextual or special connotations were noted and pulled out as illustrative quotes.

The analysis commenced in the field while the data were reviewed and corrected for accuracy and clarity. All interviews and discussions were transcribed and the transcripts typed with MSWord processing package and converted into American standard code for information interchange rich text format (RTF) files. These were coded and sorted using the Atlas.ti version 6 program.

The next level of analysis began with the review of the interview and discussion experiences with the field assistants who facilitated and recorded interviews and discussions, this time to obtain their views on the factors that inhibited or animated discussions. A more detailed analysis began with the researchers reading the transcript. During the first reading, notes were made of major concepts. A second reading utilized a system of open coding. A rereading of the texts was done to discern patterns in the ordering and clustering of themes, which provided guide on the systematic development of themes and codes used in Atlas.ti software. This process ensured inter-coder reliability and facilitated triangulation of data from discussions and interviews.

## Results

### Social demographic characteristics of the respondents

A total of 246 persons (*N* = 246) were enlisted in the study and participated in the discussions (*n* = 216) and in-depth interviews (*n* = 30). Approximately one third of this was drawn from each of the participating LGA. Respondents for the in-depth interviews were mainly 12 health workers and 18 women who delivered in less than 6 months prior to the study, 4 and 6, respectively from each LGA (Table 1). The health workers were mainly females with ages between 28 and 45 years. The women who delivered in less than 6 months before the study were aged between 20 and 31 years and were into civil service and trading.

Approximately half of the FGD participants fell into the two categories of focus group discussion participants, namely mothers and husbands of women who delivered less than 6 months before the study (see Table 2). The participants were aged between 25 to 61 years with a mean age of 39.7 years (39.7 ± 8.8 SD). A total of 112 participants were females. One hundred and fifty-one of the respondents were married, and 5 were widows while 60 were single. One hundred and thirty-four of the respondents had at least secondary education.

**Table 2:** Distribution of FGD Participants by Socio-Demographic Characteristics

Socio-demographic characteristics	Frequency (*N* = 216)	Percentage
States		
Enugu east	68	32
Enugu north	81	37
Enugu south	67	31
Groups		
Mothers of women who delivered	112	52
Husband of women who delivered	104	48
Marital status		
Married	151	60
Widow	5	2
Never married	60	28
Educational level		
No formal education/Arabic	27	12
Primary	45	21.0
Secondary	111	52
Post-secondary	33	15
Occupation		
Unemployed	61	28
Civil servant	66	31
Trader	24	11
Artisan	65	30
Age group		
25–29	29	1311
30–34	24	1516
35–39	33	17
40–44	34	27
45–49	37	
50+	59	

The FGD participants were drawn from various occupational categories and were fairly evenly distributed, with the exception of the group of traders (11.1 %). Twenty-eight percent (28.2 %) was unemployed while 30.6 percent and 30.1 percent were civil servants and artisans, respectively.

### Common health problems

The discussions opened with a review of the common health problems in the communities. A number of health problems were mentioned, though with some variations between the urban/peri urban and rural LGAs. Non communicable health problems like hypertension and cardiac problems featured more in the urban/peri urban communities. On the other hand, infectious diseases featured more in the typical rural communities. Malaria featured very prominently all the communities irrespective of the level of status of the LGA (urban, peri urban, and rural). Below are samples of the quotes to illustrate the findings.

Malaria is a common thing in this community that when we see the symptoms, the people come complaining of high fever, they will complain of cold …. Malaria is the prevalent disease condition in this community because it is a tropical area. It is an endemic area for malaria and when the people come, they will complain of high fever, cough, and loss of appetite and more so malaria will be there. So there are signs and symptoms. We treat the people for malaria. [IDI, female health worker; Enugu South]

We have different diseases like cholera, malaria and eye problems, which are very common in our community…. [Participant: FGD, husbands; Enugu South LGA]

Malaria is more because it can come in different ways until that person goes for test and they confirm it to be malaria while that person must have been taking treatment for another sickness. So there are different malaria [Participant, FGD, grandmothers, Enugu North]

### Knowledge of malaria in pregnancy

Virtually all the respondents, irrespective of community, indicated awareness of malaria. Participants in the focus group discussion sessions with grandmothers and husbands of the women who delivered recently gave graphic description of the problems of malaria in the community. The common problems faced by pregnant women included malaria, nausea, body pains, bleeding, among others. The following quote from the husband of a woman who just delivered a baby, less than 6 months preceding the survey, in a focus group discussion session in Enugu East, typifies the perception of health problems of pregnant women in the communities. According to him,

Their sickness during pregnancy includes malaria. It causes death of so many women in this community after and during the delivery. It causes the placenta to go out of its tracings. Unfortunately after delivery the person dies and leaves the baby. So many women when taken to the hospital during pregnancy their legs are swollen if the delivery is coming out. The other sickness is bleeding. The women will bleed to the extent no one can stop the blood she will now die. In some of the hospitals that would have given the person treatment and equality but what they demand is unreasonable… before the husband is able to comply she will die

Malaria was one of the common problems associated with pregnancy in the study area. According to a grandmother in a focus group discussion session in Enugu East LGA, “what I know is that pregnant women like having malaria; it is almost part of their experiences and they must go through it”. Another grandmother in Enugu North local government area enumerated how the malaria in pregnant women affects their babies. According to her,

The problem it carries is that even a baby in the womb if delivered immediately is affected. In the hospital you will hear the nurse mention different type of malaria that the baby is born with. Some of these malaria types come with death.

These quotations pointed out the preponderance of malaria among the people. The pregnant women held the belief that exposure to sun, eating oily food, and drinking bad water cause malaria. In Enugu South focus group discussion session for older mothers, different perceptions on malaria causation were put forward.

…malaria is very rampant due to dirty environment and too much staying under the sun [Participant: FGD, grandmother; Enugu South]

…most women are very dirty, their lifestyle is very dirty. They don’t cook their food well and they don’t boil their water before drinking, so when you eat food that is not well cooked and drink water that is not boiled you can have malaria. [Participant: FGD, grandmother; Enugu East]

…

In Enugu North,

…everybody says malaria is caused by mosquitoes, I agree but it is not only mosquitoes that cause malaria. There are tiny insects that come out of the dirty stream near us, when these insects bite you, you can come down with malaria. These insects are worst than mosquitoes because net cannot prevent it. [Participant: FGD, husband; Enugu North]

…malaria is rampant here because of our bad weather. At times in the morning when the weather is supposed to be cool, the sun will be so hot and if you stay outside under that sun for a long time, you will fall sick. [Participant: FGD, husband; Enugu North]

The following quote from the husband of one of the participants in Enugu South threw more light on the knowledge of how people got malaria. According to him,

Malaria is a serious ailment to all living beings. Again malaria episodes are of different types. But that of pregnant women are always as a result of lack of blood…. Groundnut is not good for a pregnant woman. Similarly, too much of fresh fish in stew is bad. Mango and orange develop malaria

There seemed to be some effects of the health education given to the women in the health centers

…we have taught them that it is only mosquitoes that can cause malaria but previously they did not know that it is only mosquito. Some of them say sun, when they eat too much oil and all those stuff. [IDI, female health worker, Enugu South]

### Prevention and treatment of malaria in pregnancy

Some of the participants held the view that malaria could not be prevented. This is reflected in a sample of quotation taken from the focus group discussion session.

Malaria is a continuous process and is everywhere as you can ask anybody. There is no protective means except bringing the hospital into this community…. Nothing can be done to stop malaria as it is a continuous process as we are looking for something to cure the malaria… a lot of malaria is man-made. [FGD: husband in Enugu South].

…there is nothing like prevention of malaria, I don’t believe malaria can be prevented when everything around you gives you malaria, will you wear net and be working around? You cannot do that, so I don’t think malaria can be prevented. [Respondent: IDI, mother; Enugu South]

Others who had used insecticide-treated bednets mentioned that they are used for preventing mosquito bites and of course malaria infections. According to a grandmother in an FGD session in Enugu North, the bednets are, “to prevent mosquito from biting them and stop malaria…”. For a number of reasons, including ignorance, lack of access, poverty, and even perceived discomfort, it is not used fully. According to the same grandmother in Enugu North,

Some of them complain that it is too hot. My daughter in law complains that it is hot and does not like staying inside it…. I have it, I tie it on the window but at night I remove it because it is hot…. (General laughter).

She further demonstrated that the lack of use is due to ignorance and poverty. In her words, “some of the pregnant women do not know about insecticide treated bednets or its benefits, if there is any and they do not have money to buy it”. In a focus group discussion session with the husbands in Enugu North, a man queried, “can one sleep under insecticide treated bednets she does not have?”

Similarly, participants in the different focus group discussion sessions indicated awareness of sulphadoxine-pyrimethamine/fansidar. However, they argued that it is not used in pregnancy because it was never prescribed by doctors. The commonly prescribed drugs for the prevention of malaria were Daraprim, which they called “Sunday-Sunday medicine” and chloroquine. In a focus group discussion session with mothers in Enugu, it was agreed that “they (health workers) gave Sunday-Sunday medicine…. They gave us chloroquine….” Chloroquine is good because doctor gave it….Doctors prescribed it….”

When asked if they know fansidar, one of the grandmothers in the focus group discussion session in Enugu East said,

I know it, but I take Sunday-Sunday. When I was pregnant I was given Sunday-Sunday…. If you are pregnant, Doctors do not prescribe Fansidar because it is strong. … it is not good for pregnant women but could be useful if you are not pregnant [Respondent: IDI, mother, Enugu East].

A grandmother in Enugu North noted that it was beyond their capacity to say how a pregnant woman with malaria should be managed. According to her,

…when I used to deliver, mosquito was not as much as they are now; malaria is too common so I don’t know the type of treatment because we are not doctors. Well, when I used to give birth, I used to go to the clinic and if I feel the symptoms of malaria and they used to give me Sunday - Sunday medicine and fruit….

A husband of one of the newly delivered women surveyed, in Enugu South, noted that,

Some of the others will go to Native Doctors, Hospital, clinic for treatment and even the people in the hospital don’t know any wrong from right. Whereas, some of the herbalists know the type of herbs to be administered, in the hospital the nurses that are there will not want to treat the pregnant women for safe deliver.

Another husband from Enugu East emphasized that,

There are traditional birth attendants that can give drugs and herbs that will subside for the temporarily, since they are not real doctor who learn about drugs proper.

A grandmother in Enugu East noted that,

Most times, for stubborn malaria, they use mix alba, essom salt, and are advised to take anaema like boiled leaves and drink.

Another grandmother from Enugu North wondered why they should bother with knowing the ways of managing malaria in pregnancy when the doctors normally dispense “Sunday-Sunday” medicine to them. According to her, “it is like that question is higher than us because we are not doctors….”

In a focus group discussion session in Enugu North with husbands, participants threw more light on how their wives sought treatment when they were ill with malaria. According to one of the participants,

…whenever she is sick, we go the chemist first and buy some drugs. If there is any dealer there we asked him what drug to buy, if not we buy the one we know. If that does not work, we shall now go to nurse (auxiliary nurse) to give her injection, when we have tried all these ones and it doesn’t work, we will now go to hospital. [Participant: FGD, husband; Enugu North]

### Barriers for ITN and IPT use

Discussions from focus group discussion sessions in Enugu South and North with the women and husbands of pregnant women stressed the point that if the services in antenatal clinic were good, the cost and distance would not be a hindrance. They emphasized the fact that,

… pregnant women like to go for ante-natal but the problem is the quality of care. Most of the nurses are not qualified for the job. When you come for ante-natal, they just stay there and be shouting at the pregnant women. When you complain of sickness, they do not give you anything; they just tell you that government did not give them drugs. This is very common with government hospitals, the private ones are better. [Respondent: IDI, woman; Enugu South]

…the roads are very bad, the pregnant women find it difficult to come out to the hospital because the transport people say that the road will spoil their buses. But if they hear that the government brought drugs, they will enter ‘okada’ (commercial motorcycle transportation) or trek to the hospital so that they can get these drugs. [Respondent: IDI, woman; Enugu South]

…my wife goes for ante-natal in a private clinic because she is more comfortable there. The environment of the clinic is clean, the nurses attend to her well, they give her ante-natal drugs and the doctor checks her every time she goes there. It is more expensive and far from our house but she will rather go there than go to government hospital and collect insults. [Participant: FGD, husband; Enugu South]

The people experienced a lot of problems with the orthodox health system, some of which included the attitude of the health workers, distance, and cost. In the alternative therefore, they sought traditional treatment. According to the husband of a recently delivered woman in Enugu South, they do not go to doctors “since the hospital is far. Traditional birth attendants, living in the village attended to them”. Another husband from Enugu North argued,

…. Even if the drugs are sent to primary health centre the Nigerian Nurses will not give you the drugs. I witnessed, from the last bednet that was given, that the Nigerian Nurses deprived the people by saying how can they sleep under the Bed Net. I don’t know how the Nigerian profess to humanitarian service as to enable them have one for mankind. We know that Nurses are the worst set of people, arrogant.

One could also trace the non-use to unavailability of these commodities across Enugu. The focus group discussions and in-depth interviews carried out during the course of this study revealed how the various participants (both men and women) and medical practitioners strongly indicated the unavailability of insecticide treated bednets, for instance.

In Enugu North focus group discussion session with husbands of pregnant women, a participant mocked:

What nets? There are no nets, they tell us that mosquito nets are available free of charge but when we ask for it they always tell us that government did not provide any net. [Participant: FGD, husband; Enugu North]

It is all a ‘419’ scam, we always hear that WHO has provided mosquito nets for the hospital and for distribution but we have never seen it. We only see it in the market and it is very expensive. I am sure the government people sell it to those traders in the market. [Participant: FGD, adult male; Enugu North]

In-depth interviews in Enugu North and Enugu East.

…those who are in charge of insecticide treated bednets distribution should increase their efforts. They are not doing enough; they need to go from house to house to give out these nets. They should also make these nets available to hospitals and health centers so that it can be given to pregnant women when they visit. [Respondent: IDI, health worker; Enugu East]

…most women that come here say that they buy insecticide treated bednets from the market. Nobody has given me any net to give to the women that come here, so I advise the women to go and buy. [Respondent: IDI, health worker; Enugu North]

Health workers interviewed noted difference in the effectiveness of the urban and rural local government areas in the distribution of intermittent presumptive treatment in pregnancy and insecticide-treated bednets than others. According to one of the health workers interviewed,

In this local government, the ministry of health gives us our drugs; in fact they distribute it to all the local governments. At a certain time, I even heard that they were giving out insecticide treated bednets to people. [Respondent: IDI, health worker; Enugu North]

I understand that the ministry of health gives drugs freely to all the local government areas and it is left for the local government to know how to distribute it to the hospitals and health centers. Even the insecticide treated bednets, I heard that the local government people were going house to house to give people nets. [Respondent: IDI, health worker; Enugu North]

For reasons of unavailability of health commodities, as captured in the preceding quotes, pregnant women prefer to go to private hospitals for antenatal care and other healthcare needs; this is largely as a result of poor conditions of government owned health facilities, lack of drugs, and hospital equipments. Focus group discussion sessions held across Enugu local government areas threw more light on how serious this problem is,

… most of these government hospitals don’t have anything; when my wife went there they could not even give her panadol. They always say that government did not give them any drug, so I decided that she should register in a private clinic even though they are expensive at least they would give her quality care [Participant: FGD, adult male; Enugu South]

If you go to our government hospitals, they are very dirty, the nurses are very rude. I even heard that they used to slap these pregnant women when they are in labor, so I did not even allow her to go there. We just went to a private hospital and registered. [Participant: FGD, adult male; Enugu North]

In these government hospitals, they give fake drugs. They give you chalk when you take it, what is happening will still be happening… [Participant: FGD, older mothers; Enugu South]

If a woman wants a safe delivery, she should just go to a private clinic. People say that government hospitals have equipment and drugs but it is a lie. When you go there they will still write drugs for you to go and buy and the equipments are not even working and you can never see doctor only rude nurses. Like now the doctors are on strike, so there is no need. [Participant: FGD, older mothers; Enugu North]

The attitude of health workers discouraged the women from going to hospital for antenatal care and even when they did, it was not done early in the pregnancy. For instance, the husband of a newly delivered woman in Enugu South mentioned that, “the normal month for this is four (4) months. But my wives treated them with herbs as they do not register in any of the hospital. I treat them with herbs.”

Women who indicated that they took sulphadoxine-pyrimethamine reported that they got it from health facility/during antenatal care visit. Others indicated that they got it from the pharmacy. The qualitative data showed that women often receive a variety of services. According to a grandmother in Enugu South,

When we were pregnant in our days, they used to cut leaves, squeeze it and give to us to drink, if the child was disturbing you she will lie still until you deliver. .... But now it is not easy, power keeps attacking them. … Women have really died on the way. Labour can be prolonged with the cord twisting around the neck of the baby.

Some doctors considered administration of intermittent presumptive treatment in pregnancy as unnecessary. They preferred to wait for the pregnant woman to come down with malaria before they treat. This fact was deduced from an in-depth interview carried out with a health worker in Enugu North local government area,

…we use paludrine as a prophylactic dose in early pregnancy. We do not really administer intermittent presumptive treatment because from my own perspective and experience, we have a lot of fake drugs. So I rather treat malaria than give prophylactic drug that would harm the baby. [Respondent: IDI, health worker; Enugu South]

## Discussion and conclusions

This study revealed different levels of awareness of malaria and how it should be managed during pregnancy. These levels of awareness were also found to reflect on actions taken to prevent malaria during pregnancy. Those who were aware used insecticide-treated bednets and intermittent presumptive treatment during pregnancy. These findings confirm findings from other studies. Studies on disease control and utilization of health promotion interventions have identified community perception as a major driver of the people’s attitude towards the interventions. Those who hold this view argue that utilization of such health interventions is only partially explained by availability, while the major decider lies in the people’s perception, which forms basis of their decision on whether or not to use the interventions [23–25]. The results of this study confirm these positions. Some scholars had also argued that assessment of the people’s perceptions is germane to the development and delivery of appropriate health interventions [26]. This study investigated people’s perceptions and attitudes towards use of health commodities designed for preventing malaria in pregnancy and showed results that corroborate earlier studies. The study indicated that pregnant women perceived high fever and general weakness as a normal sign of pregnancy. However, their perceptions of the preventive technologies affect the use of the same technologies considered safe, low cost, and effective in protecting women against malaria infection during pregnancy. This is not peculiar to Nigeria. A study by Njama, Dorsey, Guwatudde, Kigonya, Greenhouse, Musisi, and Kamya (2003) in Kampala city also indicated that 90 % of the care givers knew that mosquitoes cause malaria although they equally indicated other perceived causes such as drinking unboiled water and respiratory illnesses [27].

Despite awareness of the causes and implication of malaria in pregnancy, insecticide-treated nets (ITN) and intermittent preventive treatment (IPT) as preventive or treatment measure, and the health facilities to be the first place to go at the onset of illness, misconceptions and poor attitude of health workers towards patients affected the uptake of interventions related to malaria in pregnancy. Antenatal care at health facilities is key in the delivery of malaria in pregnancy prevention interventions [28–31]. However, the women seemed discouraged with the health system and rather go for alternative health care. Some studies have highlighted the social, cultural, and health system constraints to the use of health facilities [30]. Health workers’ attitudes and behavior towards pregnant women and attitudes towards specific services offered can also potentially deter women from accessing ANC at health facilities [32].

The limitation of this study is the inability to generalize the results to larger populations. It is a qualitative study in which the inclusion of participants was not based on any statistical consideration. Randomization was only limited to the selected of communities. Consideration was not given to ensure the inclusion of all necessary parameters of the larger population. Another limitation is the inability to make statistical conclusions on the occurrence of the perception. However, this study provides data that could form the basis of a study using a more rigorous statistical and mixed method design. It provides a range of perceptions that would form used categories in developing structured data capture tool for quantification in future.

The findings here confirm the theoretical basis of this study. Based on the health belief model, one can conclude that pregnant women in Enugu State would adopt malaria preventive measures if these measures were available and if they are sufficiently educated on the values of these measures. However, the health workers who should educate the women to use these intervention are themselves short of world expected standard. The health workers also need to be trained on appropriate communication skills so as to act as motivators, rather than inhibitors. It is thus recommended that stakeholders ensure the availability of the commodities in adequate quantity and quality with health education to promote effective access among pregnant women as well as target health workers to create more friendly environment for the clients.
